# Goal-Based Binding of Irrelevant Stimulus Features for Action Slips

**DOI:** 10.1027/1618-3169/a000525

**Published:** 2021-12-17

**Authors:** Anna Foerster, Klaus Rothermund, Juhi Jayesh Parmar, Birte Moeller, Christian Frings, Roland Pfister

**Affiliations:** ^1^ Department of Psychology III, Julius-Maximilians-Universität of Würzburg, Germany; ^2^ General Psychology II, University of Jena, Germany; ^3^ Department of Cognitive Psychology, University of Trier, Germany

**Keywords:** action control, error processing, binding and retrieval, distractor–response binding

## Abstract

**Abstract.** Binding between representations of stimuli and actions and later retrieval of these compounds provide efficient shortcuts in action control. Recent observations indicate that these mechanisms are not only effective when action episodes go as planned, but they also seem to be at play when actions go awry. Moreover, the human cognitive system even corrects traces of error commission on the fly because it binds the intended but not actually executed response to concurrent task-relevant stimuli, thus enabling retrieval of a correct, but not actually executed response when encountering the stimulus again. However, a plausible alternative interpretation of this finding is that error commission triggers selective strengthening of the instructed stimulus–response mapping instead, thus promoting its efficient application in the future. The experiment presented here makes an unequivocal case for episodic binding and retrieval in erroneous action episodes by showing binding between task-irrelevant stimuli and correct responses.


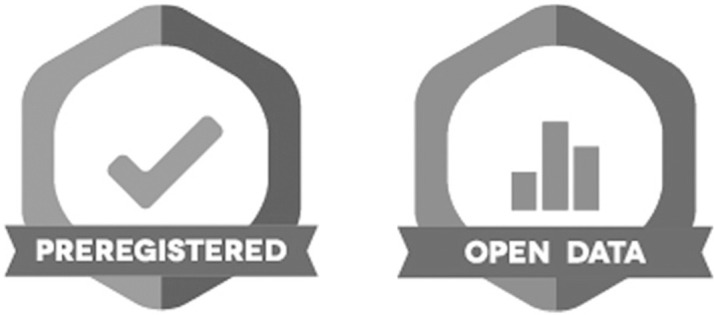


Human action control relies on binding mechanisms that integrate representations of stimuli, responses, and effects of an action episode (e.g., Frings et al., 2020; Hommel, 2004; Moeller et al., 2019). This integration process facilitates future actions because reactivating any element of an existing compound can retrieve all other elements that were bound to it, which expedites action selection and planning (for corresponding long-term associations, see Logan, 1988).

Despite widespread agreement that binding and retrieval are ubiquitous in human action control, current theories are underspecified as to when binding actually occurs. Following the notion that binding and retrieval support efficient behavior, previous accounts have proposed binding to rely on the evaluation of an action episode as successful (*success-based binding*; Hommel, 2005). Such an architecture would ensure that previously inefficient or erroneous actions are not retrieved when re-encountering a similar situation. Recent findings, however, challenge this assumption and point to a strikingly adaptive property of episodic binding and retrieval (Foerster et al., in press). These findings indicate that the human cognitive system corrects erroneous action representations by binding representations of task-relevant stimuli to the intended correct responses (*goal-based S*_*rel*_*-R*_*cor*_ *binding*), whereas the actually executed erroneous responses enter bindings with irrelevant effects that they produce (*R*_*err*_*-E*_*irr*_ *binding through coactivation*). More precisely, this study observed that repeating a target stimulus across two successive trials facilitated performance of the correct response following an erroneous action episode, whereas presenting a previous effect facilitated performance of the response that had been made in error. Binding and retrieval thus seem to be tuned toward contingencies between actions and action-triggered changes in the agent’s environment. For example, if someone is driving a car and an obstacle on the road forces a sudden lane shift, the driver should activate the indicator by pushing the left lever next to the steering wheel. In the heat of the moment, the driver might end up pushing the right lever instead, which would trigger the windshield wiper instead. Episodic binding would create compounds between obstacles on the road ahead and pushing the left lever (i.e., the intended but not executed correct response) as well as between pushing the right lever (i.e., the actually executed erroneous action) and the moving windshield wipers. This ensures that re-encountering situations would retrieve intended correct responses although actions are represented with their effects on the environment, regardless of whether the action was appropriate or inappropriate, mirroring the adaptive properties of higher-level processes during error-based learning (Mohr et al., 2018).

The current experiment rules out a critical alternative explanation of the findings on goal-based *S*_*rel*_-*R*_*cor*_ binding and retrieval for erroneous action episodes (Foerster et al., in press). Instead of binding the representation of an intended correct response to features of a task-relevant stimulus, one could also suspect that agents strengthen the stimulus–response mapping rule that they had just violated inadvertently. That is, after delivering a wrong response, agents specifically strengthen the instructed mapping rule of the current stimulus. Coming back to the car example, drivers would retrieve what they have previously learnt about how to turn on the indicator when they see an obstacle on the road ahead. Higher accessibility of this mapping rule compared to all other mapping rules would facilitate correct response repetitions for stimulus repetitions compared to stimulus changes. Such strategies might be expected to boost performance in light of accumulating evidence for a consistent impact even of merely instructed mapping rules (e.g., Braem et al., 2019; Cohen-Kdoshay & Meiran, 2009; Kunde et al., 2003; Meiran et al., 2015; Pfeuffer et al., 2017; Wenke et al., 2007), which is possibly mediated by the formation of efficient action triggers (Kiesel et al., 2007) or implementation intentions (“if *S*_*rel*_, then *R*_*cor*_”; Gollwitzer, 1999). This account would thus assume that previous evidence for goal-based binding mirrors covert strengthening of a mapping rule rather than actual binding (for a related discussion in the literature on prospective memory, see Streeper & Bugg, 2021).

One way to disentangle the contributions of rule strengthening and binding is to investigate binding and retrieval effects of task-irrelevant stimuli.^1^ Observing performance facilitation of an intended correct but not actually executed response in the face of repetitions of the irrelevant stimulus (relative to changes of the irrelevant stimulus) would make a strong case for goal-based binding because the irrelevant stimulus is not part of any mapping rule and simply co-occurs with all targets.^2^ For correct action episodes, binding and retrieval effects between stimuli and responses emerge even if stimuli are completely uninformative for the successful completion of the task (e.g., Giesen et al., 2012; Moeller & Frings, 2014). For example, the obstacle on the road might have been a red car. Although, for indicating the lane change, it is completely irrelevant that the car is red, the color feature would still be bound together with the response of successfully turning on the indicator. Goal-based binding for errors would predict that similar binding and retrieval effects also arise after action slips. At the same time, there should not be an existing representation of a rule that specifies that drivers should indicate by operating the left lever whenever they see red on the road ahead.

The absence of task rules for irrelevant stimuli also means that goal-based binding for action slips would not be adaptive because it does not systematically bias behavior toward future success – at least when not assuming positive correlations between relevant and irrelevant stimuli. This perspective suggests that intentions might not matter for binding of irrelevant stimuli if binding occurred in this context. Instead, there might be *S*_*irr*_-*R*_*err*_ binding through coactivation, equivalent to binding of erroneous responses and their effects that were also independent of any mapping rules (*R*_*err*_-*E*_*irr*_ binding through coactivation; Foerster et al., in press). Translated to the car example, the color red would be associated with erroneously pushing the right lever that controls the windshield wipers.

Finally, considering that neither goal-based *S*_*irr*_-*R*_*cor*_ binding nor *S*_*irr*_-*R*_*err*_ binding through coactivation would be truly adaptive because both reflect an incidental co-occurrence of an irrelevant stimulus with an executed or required response, the cognitive system might randomly employ either of them, resulting in null effects. Such a result would also be in line with the traditional stance in the literature of a success-based binding mechanism that is only effective in correct action episodes (Hommel, 2005). In case of random bindings, however, variance of performance should be higher for repetitions rather than changes of the irrelevant stimulus. Random retrieval of either the correct or the erroneous response from an irrelevant stimulus should produce facilitation or interference, depending on the response relation between successive trials. No such increase in variance should emerge if binding and retrieval are cancelled for erroneous responses, as suggested by the success-based binding account.

In a nutshell, we examined whether irrelevant stimuli enter bindings with either the correct (i.e., goal-based *S*_*irr*_-*R*_*cor*_ binding) or the erroneous response (i.e., *S*_*irr*_-*R*_*err*_ binding through coactivation) in erroneous action episodes. We tested our predictions via sequential analyses in a speeded choice-reaction time task (e.g., Frings et al., 2007; Giesen & Rothermund, 2014b; Moeller et al., 2016). Participants responded to the identity of target letters with button presses in a 6:2 mapping (see Figure 1). Target letters were presented within one of two irrelevant color patches. Therefore, we could analyze performance as a function of whether the preceding response was correct or a commission error and whether irrelevant stimuli (color patches) and correct responses repeated or changed^3^ from the preceding to the current trial. Figure 2A shows corresponding model predictions. We expected typical *S*_*irr*_-*R*_*cor*_ binding and retrieval effects in correct action episodes to emerge as an interaction between the sequence of irrelevant stimuli and correct responses. This interaction should reflect stronger benefits of repeating than changing irrelevant stimuli for correct response repetitions relative to correct response changes because a repetition of irrelevant stimuli should retrieve the previously bound correct response (see Figure 2B). Goal-based binding predicts a similar effect after erroneous responses. Binding through coactivation would predict the opposite pattern, that is, larger costs of repeating than changing irrelevant stimuli for correct response repetitions relative to correct response changes, because a repetition of irrelevant stimuli would retrieve the previously bound erroneous response.

**Figure 1:**
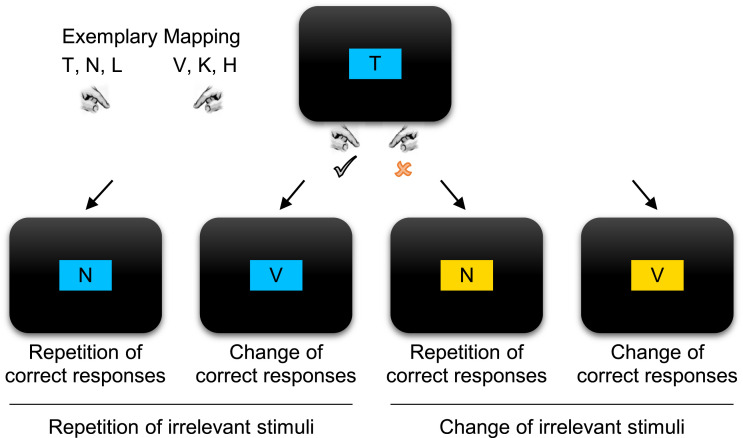
Experimental conditions. Participants had to classify the target letter within 600 ms with a left response (mapped to stimuli *T, N*, and *L* in this example) or a right response (here: *V, K*, and *H*) while ignoring the irrelevant color patch (see colored version in the online article). Stimuli are not drawn to scale. Across successive trials, irrelevant stimulus colors could either repeat or change; the same held true for correct responses that either repeated or changed depending on the identity of target letters

**Figure 2:**
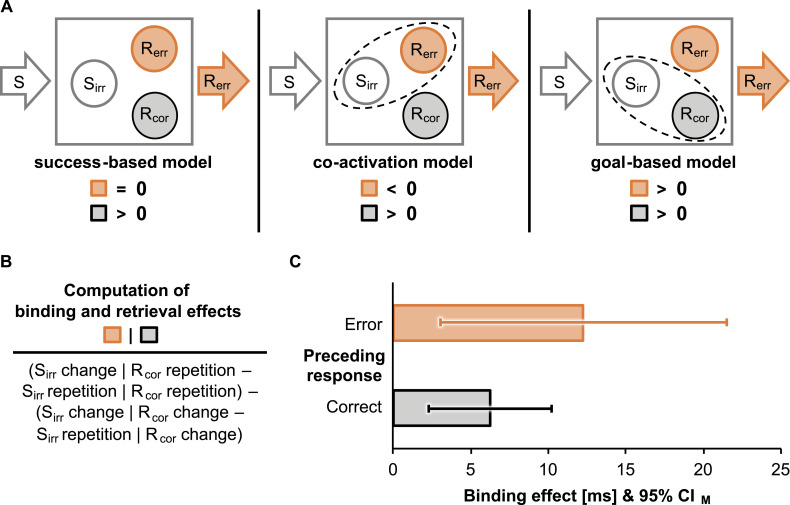
Model predictions and binding and retrieval effects (see colored version in the online article). (Panel A) Potential mechanisms for binding between representations of an irrelevant stimulus (*S*_*irr*_), an erroneous response (*R*_*err*_), and a correct response (*R*_*cor*_) when agents commit an error (filled orange arrow) in responding to stimuli (relevant and irrelevant; white arrow). Binding between these representations is illustrated by dashed outlines, supplemented by model predictions (see Panel B for the computation of empirical effects). For correct action episodes, all models predict binding of the correct response and therefore positive binding and retrieval effects (bottom grey squares). For erroneous action episodes (top orange squares), the success-based model predicts no binding and therefore nonsignificant effects, and the coactivation model predicts binding of the erroneous response and therefore negative effects, whereas the goal-based model predicts binding of the intended correct response and therefore positive effects. (Panel B) Binding and retrieval effects for erroneous action episodes (left orange square) and correct action episodes (right grey square) were computed as the difference in stimulus repetition effects between repetitions and changes of the correct response. (Panel C) Mean binding and retrieval effects following erroneous (top orange bar) and correct responses (bottom grey bar). Error bars represent the 95% CIs of the mean (*CI*_*M*_).

## Methods

### Participants

For sample size calculations, we relied on data from our laboratory on goal-based binding of relevant stimulus information for erroneous action episodes (*d*_*z*_ ≥ 0.77; i.e., the comparison of correct response repetitions between target repetitions and target changes). A sample of 24 participants has a power of 95% to detect this effect size in a two-tailed paired-samples test with an α level of 5% (calculated with the power.t.test function in R version 3.3.3). We pre-registered this experiment (https://osf.io/32hjg, Foerster et al., 2021) and decided to collect data from 48 participants to compensate for potential data exclusions (see below) and potentially smaller effects due to the present focus on task-irrelevant stimuli. We had to exclude one participant because they aborted the experiment prematurely.

### Apparatus and Stimuli

Participants conducted the experiment alone in a room on a setup with a 24″ screen (display resolution; 1,920 × 1,080 pixels; refresh rate: 100 Hz) and a standard German QWERTZ keyboard. They pressed *F* and *J* with their left and right index fingers. One key mapped to letters *T*, *N*, and *L*, and the other key mapped to letters *V*, *K*, and *H*. We counterbalanced the assignment of letter triplets to the two response keys across participants. Target letters appeared centrally in size 22 pt. and black font against a task-irrelevant color patch (yellow vs. blue) that was 480 pixels in width and 270 pixels in height.

### Procedure

Participants received written instructions on which keys to use and on the letter–key mapping. We also informed them about the irrelevant color patches, specifying that these would not map to any response alternative and that participants should do their best to not get distracted by these stimuli. Participants could repeat the instructions if they wanted to.

Each trial began with a fixation cross for 750 ms. Then, a target letter and irrelevant color patch appeared on screen until participants responded or 600 ms passed. Participants received performance-contingent feedback after each trial in the practice block (1,000 ms; translated from German: *Good!* in green font for correct responses; *Too slow!* in red font if no response had been registered within 600 ms after stimulus onset; and *Wrong!* in red font for wrong keypresses with the instructed keys, i.e., commission errors, or any other key, i.e., random keypress). Feedback was only given after omission errors (i.e., no response within 600 ms) in the experimental blocks. We also registered late responses during fixation. Although we did not consider these for feedback, their registration was necessary for data treatment. We opted against feedback for both correct responses and false keypresses in experimental blocks to create equivalent procedures for a fair comparison between correct and erroneous trials. Instead, we provided aggregated information about the number of omission and commission errors as well as mean correct response times (RTs) after each block.

Participants went through 112 trials per block for a total of one practice block and 19 experimental blocks. We determined the target letter and the irrelevant color randomly for the first trial of each block. For all remaining trials, we aimed at an equal distribution of the four experimental conditions, that is, sequence of irrelevant stimuli (repetition vs. change) × sequence of correct responses (repetition vs. change), in action episodes with a commission error even if commission errors would occur only rarely. Therefore, we created two separate arrays with a random sequence of two instances of each of the four individual condition sequences. That is, each array had the same eight elements. Whenever participants committed an error (i.e., pressed the left response key when the right response key would have been appropriate, or vice versa), the condition sequence was determined via one array, whereas it was determined via the second array in all other cases. Each array was re-set and randomized as soon as all elements had been drawn from it. The combination of condition sequence and color identity of the preceding trial then determined the color identity of the current trial. The condition sequence determined the relevant target triplet, of which one target was drawn randomly.

### Data Treatment and Analysis

We excluded the first trial of each block because these trials did not allow for sequential analyses of irrelevant stimuli and responses. We further removed target repetition trials (16.8%) from our analyses to control for potential binding and retrieval effects of the target with either the irrelevant stimulus or the response (Giesen & Rothermund, 2014a). We selected trial sequences with a correct response or a commission error in the preceding trial, excluding 4.3% random keypresses or late responses and 3.7% omissions in the preceding trial. For this selection of trials, we computed the percentage of commission and omission errors for statistical analyses (number of commission or omission errors/sum of the number of correct trials and commission or omission errors). We further selected trial sequences with a correct response in the current trial for all RT analyses. We also excluded trials with RTs that deviated more than 2.5 *SD*s from their corresponding cell mean as outliers (1.3%). After these preprocessing steps, we excluded two participants from all statistical analyses because their data came with less than 10 usable trials in at least one cell of the RT analysis, resulting in a final sample size of 45 participants (for descriptive statistics on the number of trials available for analysis, see Table 1).

**Table 1:** Descriptive data

Preceding response	Sequence of irrelevant stimuli	Sequence of correct responses	RT (ms)	Trials for RT analysis	Commission errors (%)	Omission errors (%)
Correct	Repetition	Repetition	420 (46)	223 (53)	13.1 (7.5)	3.8 (3.1)
		Change	443 (40)	326 (101)	18.4 (14.8)	5.3 (6.7)
	Change	Repetition	422 (52)	224 (53)	14.1 (7.6)	3.8 (3.3)
		Change	439 (38)	332 (100)	16.6 (14.6)	4.9 (6.4)
Error	Repetition	Repetition	430 (48)	31 (16)	16.7 (13.9)	7.1 (6.4)
		Change	441 (55)	45 (22)	18.9 (14.1)	6.3 (7.1)
	Change	Repetition	435 (44)	31 (17)	16.6 (13.9)	7.7 (6.5)
		Change	433 (53)	46 (21)	17.0 (16.2)	6.5 (8.2)
*Note*. *M* and *SD*s (within parentheses) of response times (RTs), number of trials for the analysis of RTs as well as the percentage of commission and omission errors for each combination of preceding accuracy, sequence of irrelevant stimuli and sequence of correct responses.						

We analyzed RTs in a 2 × 2 × 2 repeated measures ANOVA with the factors preceding response (correct vs. commission error), sequence of irrelevant stimuli (repetition vs. change), and sequence of correct responses (repetition vs. change). A significant three-way interaction was followed up in separate 2 × 2 ANOVAs for preceding correct and erroneous responses. Significant two-way interactions were further explored in two-tailed paired-samples *t*-tests. We also employed these tests for the percentage of commission and omission errors to reveal potential speed accuracy trade-offs. We further announced similar secondary analyses for the variability of RT and differences in response duration between responses of successive trials in our pre-registration but do not report these analyses in detail here for brevity. In short, we did not find binding and retrieval effects in either measure (nonsignificant interactions of sequence of irrelevant stimuli × sequence of correct responses and three-way interactions, *F*s < 1). By contrast, we found evidence for binding and retrieval in the variability of RTs after correct and erroneous action episodes for relevant stimuli (Foerster et al, in press; see also for an introduction and discussion of these measures). The full analysis of these measures is included in our analysis syntax (https://osf.io/nsqu7, Foerster et al., 2021).

## Results

**Figure A1:**
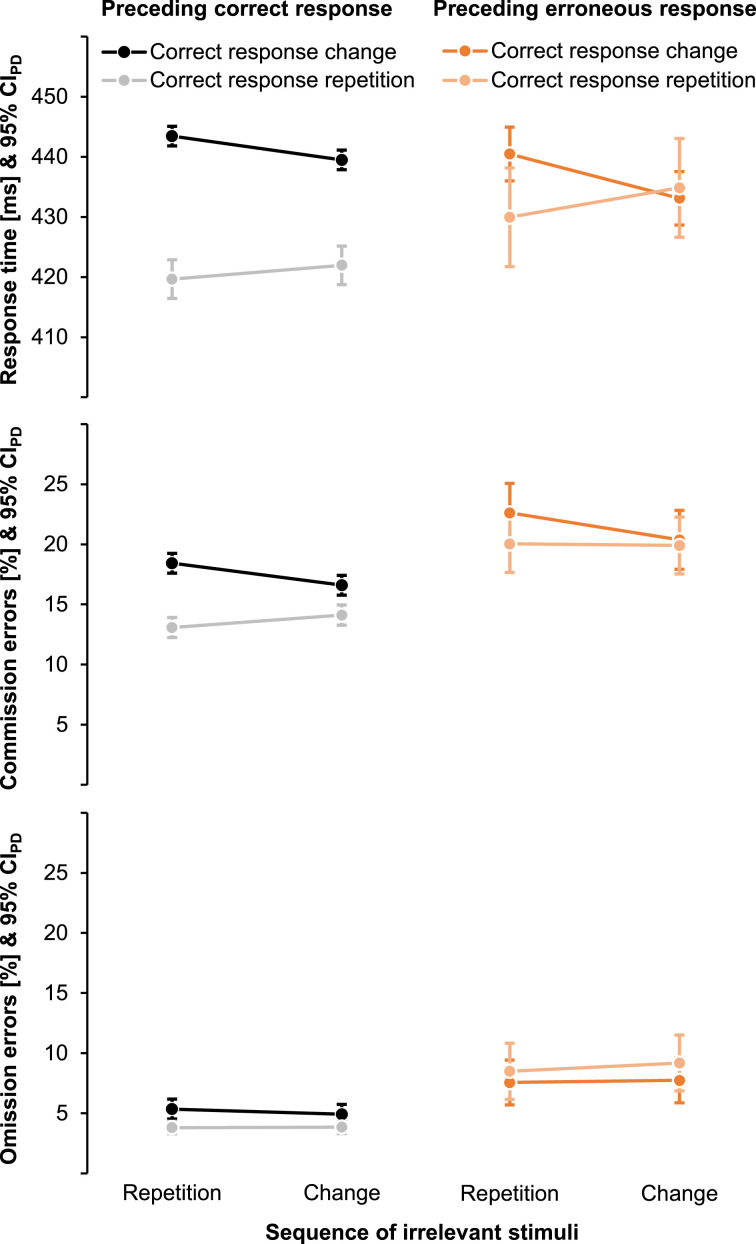
Full overview of the results. Response times (top), commission errors (middle) and omission errors (bottom) as a function of preceding response (correct vs. commission error), sequence of irrelevant stimuli (repetition vs. change), and sequence of correct responses (repetition vs. change). Error bars represent the 95% confidence intervals of the paired differences (CIPD), computed separately for correct response repetitions and changes (Pfister & Janczyk, 2013). See colored version in the online article.

All data and analyses are publicly available (https://osf.io/nsqu7, Foerster et al., 2021). Table 1 and Figure A1 in the Appendix provide a full overview of the descriptive data for each of the dependent variables separated by experimental conditions.

### Response Times

Changes of correct responses were slower than repetitions, *F*(1, 44) = 12.66, *p* = .001, η_*p*_^2^ = .22, whereas none of the remaining main effects were significant, *F*s(1, 44) ≤ 1.36, *p* ≥ .251, η_*p*_^2^ ≤ .03. Preceding accuracy and the sequence of irrelevant stimuli did not interact, *F* < 1*.* However, preceding accuracy interacted with the sequence of correct responses, *F*(1, 44) = 4.54, *p* = .039, η_*p*_^2^ = .09, because benefits of repeating rather than changing correct responses were only evident after correct responses (*M* = 20.67, *SD* = 28.33), *t*(44) = 4.90, *p* < .001, *d*_*z*_ = 0.73, but not after commission errors (*M* = 4.39 ms, *SD* = 40.34 ms), *t*(44) = 0.73, *p* = .470, *d*_*z*_ = 0.11. Crucially, the two-way interaction of the sequence of irrelevant stimuli and correct responses was significant (see Figure 2C), *F*(1, 44) = 21.07, *p* < .001, η_*p*_^2^ = .32, indicating typical binding and retrieval effects as repetitions of the correct response showed a nonsignificant trend to be faster with repetitions than changes of irrelevant stimuli (*M* = 3.60 ms, *SD* = 12.30 ms), *t*(44) = 1.96, *p* = .056, *d*_*z*_ = 0.29, while changes of the correct response were significantly slower for repetitions rather than changes of irrelevant stimuli (*M* = −5.68 ms, *SD* = 7.91 ms), *t*(44) = −4.81, *p* < .001, *d*_*z*_ = −0.72. Binding and retrieval effects did not differ significantly between correct and erroneous action episodes, as suggested by a nonsignificant three-way interaction, *F*(1, 44) = 1.08, *p* = .304, η_*p*_^2^ = .02.

### Commission Errors

Erroneous responses compared to correct responses in the preceding trial increased the percentage of commission errors, *F*(1, 44) = 4.86, *p* = .033, η_*p*_^2^ = .10. There was a nonsignificant trend toward more commission errors in sequences with a repetition compared to a change of irrelevant stimuli, *F*(1, 44) = 3.13, *p* = .084, η_*p*_^2^ = .07. The main effect of the sequence of correct responses was not significant, *F*(1, 44) = 2.77, *p* = .103, η_*p*_^2^ = .06. Crucially, the interaction of the sequence of irrelevant stimuli and correct responses was significant, *F*(1, 44) = 10.22, *p* = .003, η_*p*_^2^ = .19. Typical binding and retrieval effects emerged in that there was no effect of the sequence of irrelevant stimuli if both trials afforded the same correct response (*M* = 0.46%, *SD* = 3.31%), *t*(44) = 0.94, *p* = .354, *d*_*z*_ = 0.14, whereas trials with repetitions of irrelevant stimuli were more error-prone than trials with changes of irrelevant stimuli if the two succeeding trials afforded a change of the correct response (*M* = −1.85%, *SD* = 3.84%), *t*(44) = −3.23, *p* = .002, *d*_*z*_ = −0.48. None of the remaining two-way interactions, nor the three-way interaction, were significant, *F*s ≤ 1.

### Omission Errors

Erroneous responses in the preceding trial also increased the percentage of omission errors compared to correct responses, *F*(1, 44) = 33.89, *p* < .001, η_*p*_^2^ = .44. None of the other main effects were significant, *F*s < 1. There was a significant interaction between preceding accuracy and the sequence of correct responses, *F*(1, 44) = 6.67, *p* = .013, η_*p*_^2^ = .13, because sequences that afforded the same correct response were more accurate than sequences where the correct response changed after a preceding correct response (*M* = 1.30%, *SD* = 4.09%), *t*(44) = 2.14, *p* = .038, *d*_*z*_ = 0.32, whereas there was no effect of correct response sequence after a preceding erroneous response (*M* = −0.99%, *SD* = 5.54%), *t*(44) = −1.20, *p* = .235, *d*_*z*_ = −0.18. No other interactions were significant, *F*s < 1.

## Discussion

The current experiment addressed binding and retrieval following action slips. Participants performed a speeded choice reaction task, and we investigated whether sequential analyses of performance data would support the notion that goal-based binding for action slips also encompasses task-irrelevant stimuli. Results supported this notion by showing goal-based binding of task-irrelevant stimuli to the intended correct response instead of the erroneous response that had been executed. This finding makes a strong case that episodic binding is indeed at the heart of previous observations on binding and retrieval for action slips (Foerster et al., in press), thus ruling out task-set strengthening as an alternative explanation. Together, the two studies demonstrate that both relevant and irrelevant stimuli enter bindings with the intended correct response instead of the erroneous response, whereas *R-E* bindings incorporate the executed erroneous response.

How is it possible that both binding mechanisms operate in action slips? Previous research already demonstrated that *S*_*irr*_-*R* and *R*-*E* bindings of the same action episode can exist independently without *S*_*irr*_-*E* bindings (Moeller et al., 2019). In this study and the current one, relevant or irrelevant stimuli were present during the planning of correct and erroneous responses until response registration, whereas effects only occurred after responding without providing any information on accuracy. The time course, the perceived structure of events, or the inferred causality might, therefore, be crucial determinants of the type of binding. Goal-based binding might be evident in the course of preparing and executing a response, whereas binding through coactivation might take over in the aftermath of a response during the monitoring of actions and effects. Therefore, it might be worthwhile to investigate systematically whether binding for action slips depends on whether stimuli are mostly present before, during, or after the response.

Expanding the scope of binding in erroneous action episodes to irrelevant stimuli further supports the notion that binding and retrieval do not hinge on the evaluation of an action episode as successful (Hommel, 2005). The current study even shows that the quality and quantity of binding can be equivalent in correct and erroneous action episodes. By contrast, *S*_*rel*_-*R*_*cor*_ binding and retrieval effects were markedly weaker for action slips than for correct responses (Foerster et al., in press), with large effect sizes for correct action episodes. The current experiment observed medium-sized binding and retrieval effects for correct and erroneous action episodes alike so that *S*_*irr*_-*R*_*cor*_ binding appears to be generally weaker than *S*_*rel*_-*R*_*cor*_ binding. *S*_*irr*_-*R*_*cor*_ bindings might incorporate mostly abstract features, as, for example, left response, whereas *S*_*rel*_-*R*_*cor*_ bindings might also rely on experience-based features, as, for example, tactile feedback. Speculatively, this distinction might be rooted in knowledge about existing task rules on the one hand and concurrent evidence collection about potential contingencies on the other hand. Whereas *S*_*rel*_-*R*_*cor*_ bindings are in line with existing task rules whereby comprehensive binding of all performance-relevant features would support successful adherence to these rules, *S*_*irr*_-*R*_*cor*_ bindings do not improve task performance, but they might still be prerequisites for detecting potential contingencies between these features. For the detection of these contingencies, the incorporation of abstract features might be sufficient without an additional advantage of binding experience-based features. Crucially, abstract features of the intended correct response would be equally available for action episodes where agents execute the correct response and instances where they commit an error. By contrast, agents would have to rely on a prediction about the sensory feedback of the intended correct response if they committed an error, resulting in weaker *S*_*rel*_-*R*_*cor*_ bindings. Additional investigation on the interplay and incorporation of different available event features therefore seems to be a promising step forward in understanding binding and retrieval in general.

This study contributes another piece of evidence to the argument that binding and retrieval are a universal part of human action control. These mechanisms provide an integrated representation of perception and action that considers not only what actually happened but also what agents intended to do.

## Footnotes

^1^
Task-irrelevant stimuli are often referred to as distractors. We opted for a neutral connotation here to emphasize that our stimuli did not overlap with characteristics of task-relevant stimuli or responses, as is the case, for example, in conflict tasks. This terminology further emphasizes that seemingly distinct experimental setups, such as those addressing distractors as compared to contexts, might eventually address the same mechanisms.

^2^
The described alternative interpretation might be reframed as proposing a strengthening of an existing memory trace after an error. Such a correction process has been recently implemented in formal models of binding and retrieval (Schmidt et al., 2020). This perspective, however, would not predict goal-based *S*_*irr*_-*R*_*cor*_ binding and retrieval effects as probed for in the present study.

^3^
Note that this factor indicates whether the targets of the preceding and of the current trial required the same or a different response, irrespective of the actual response in these trials.

## Notes

**Open Data:** The preregistration (https://osf.io/32hjg), analysis syntax, and data underlying the findings reported in the article (https://osf.io/nsqu7) are publicly available. The experiment and analyses were executed exactly as pre-registered (Foerster et al., 2021).
